# Hyperinsulinemia Impairs Clathrin-Mediated Endocytosis of the Insulin Receptor and Activation of Endothelial Nitric Oxide Synthase in Brain Endothelial Cells

**DOI:** 10.3390/ijms241914670

**Published:** 2023-09-28

**Authors:** Stephanie G. DiLucia, B. Jacob Kendrick, Catrina Sims-Robinson

**Affiliations:** 1 Department of Neurology, Medical University of South Carolina, Charleston, SC 29425, USA; dilucia@musc.edu; 2 Ralph H. Johnson VA Medical Center, Charleston, SC 29401, USA; 3 Flow Cytometry and Cell Sorting Shared Resource, Hollings Cancer Center, Medical University of South Carolina, Charleston, SC 29425, USA; kendricb@musc.edu

**Keywords:** brain insulin transport, endosomes, insulin resistance, insulin signaling, internalization, obesity, transcytosis

## Abstract

Adequate perfusion of cerebral tissues, which is necessary for the preservation of optimal brain health, depends on insulin signaling within brain endothelial cells. Proper insulin signaling relies on the regulated internalization of insulin bound to the insulin receptor, a process which is disrupted by hyperinsulinemia via an unknown mechanism. Thus, the goal of this study was to characterize the impact of hyperinsulinemia on the regulation of molecular targets involved in cerebral blood flow and insulin receptor internalization into brain endothelial cells. The phosphorylation of molecular targets associated with cerebral blood flow and insulin receptor internalization was assessed in hyperinsulinemic brain endothelial cells. Insulin receptor uptake into cells was also examined in the setting of endocytosis blockade. Our data demonstrate that hyperinsulinemia impairs the activation of endothelial nitric oxide synthase. These data correspond with an impairment in clathrin-mediated endocytosis of the insulin receptor and dysregulated phosphorylation of key internalization effectors. We conclude that hyperinsulinemia alters the phosphorylation of molecular targets involved in clathrin-mediated endocytosis, disrupts signaling through the insulin receptor, and hinders the capacity for blood flow regulation by brain endothelial cells.

## 1. Introduction

Obesity affects over 650 million adults and 340 million children worldwide, with most recent predictions estimating that over half of the world’s population will be obese by the year 2035 [1,2]. In the United States, age-adjusted obesity prevalence increased amongst adults by ~8% between 2012 and 2018 alone [3]. Obesity is associated with insulin resistance, dyslipidemia, hyperglycemia, and hypertension [4]. Thus, it serves as a notable vascular risk factor and has been associated with both macro- and micro-vascular disease [1]. Obesity is strongly associated with vascular contributions to cognitive impairment and dementia (VCID), a heterogenous spectrum of disorders characterized by cognitive decline in the setting of cardio- or cerebrovascular disease [5,6]. While this association has been well characterized [7], the distinct biologic mechanisms connecting obesity and VCID remain elusive. Given the stark rise in obesity rates within an aging population [8], investigation into this pathophysiologic link constitutes an active and increasingly important area of research.

Excess consumption of energy-dense foods and physical inactivity are among the leading contributors to the development of obesity [9]. In cases of nutritional excess, the pancreas amplifies the production and release of insulin, a hormone necessary for glucose uptake into cells and tissues, thus resulting in high circulating insulin loads (hyperinsulinemia). Superfluous insulin availability promotes desensitization of insulin receptors located in tissues and vascular beds over time, thereby reducing receptor functionality and hindering proper cellular function [10,11]. As such, hyperinsulinemia-induced insulin resistance is a finding commonly seen in obese individuals [11].

Insulin action within the vasculature is important for brain insulin availability [12] and blood flow regulation [13]. Furthermore, brain insulin is important for proper cognitive function; however, there is debate over how insulin reaches the brain [12]. The predominant theory is that insulin is transported into the brain via a saturable, receptor-mediated event [14,15,16]; however, the role of the insulin receptor in endothelial transcytosis into the brain remains controversial [14,17,18]. Notwithstanding, insulin is additionally involved in vasoregulation. Insulin signaling stimulates endothelial cells to produce nitric oxide, a potent vasodilator, thereby allowing for proper blood flow to, and adequate perfusion of, cerebral tissues [13,19]. Insufficient cerebral blood flow is associated with both hyperinsulinemia-induced insulin resistance and cognitive decline [20,21,22]. Thus, we hypothesize that endothelial dysfunction following hyperinsulinemia constitutes a pathophysiologic link between obesity and cognitive decline [7].

Internalization of the insulin–insulin receptor complex is necessary for appropriate signal propagation [23] and, potentially, endothelial transcytosis [15]. In the context of insulin signaling, insulin receptor internalization and subsequent turnover is essential for controlling the sensitivity and duration of the insulin response [23]. Insulin receptor internalization has been studied at length in peripheral tissues and is known to occur via receptor-mediated endocytosis. Interestingly, how this internalization occurs differs on a tissue-specific basis, with clathrin- and caveolin-mediated mechanisms constituting the two identified pathways [24,25,26]. The mechanism of insulin uptake into vascular endothelial cells has been further proven to differ on a regional basis [27,28], highlighting the need to understand this process at the level of the brain microvascular endothelial cell. 

We have previously observed an increase in membrane-bound insulin receptors in hyperinsulinemic brain endothelial cells, suggesting that hyperinsulinemia-induced insulin resistance disrupts insulin receptor internalization [29]. Thus, in the present study, we aim to clarify how insulin receptors are internalized into brain endothelial cells, why this process is seemingly perturbed by hyperinsulinemia, and what are the downstream implications to endothelial function. To accomplish our goal, we employed naïve and hyperinsulinemic primary mouse brain microvascular endothelial cells to (1) characterize endothelial nitric oxide synthase activation, (2) assess insulin receptor internalization following pharmacologic inhibition of clathrin- or caveolin-mediated internalization, and (3) evaluate the role of insulin signaling in receptor internalization.

## 2. Results

### 2.1. Hyperinsulinemia Impairs Endothelial Nitric Oxide Synthase in Microvascular Brain Endothelial Cells (MBECs) following Acute Insulin Stimulation

**Figure 1:**
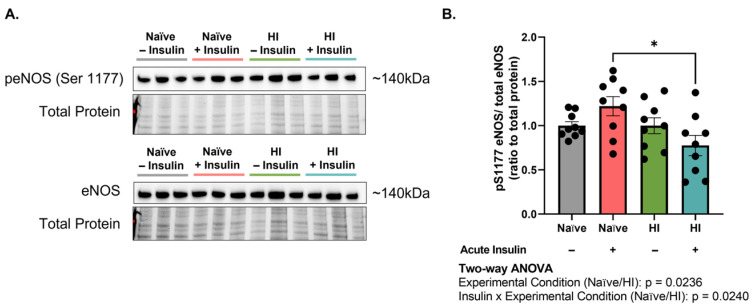
Acute insulin stimulation of hyperinsulinemic brain endothelial cells impairs nitric oxide production capacity. (**A**) Western immunoblot images and (**B**) densitometric quantification of endothelial nitric oxide synthase phosphorylated at serine 1177 (peNOS^S1177^) in relation to total endothelial nitric oxide synthase (eNOS). Chemiluminescent signals were normalized to total protein depicted in the stain-free gel. Groups shown in representative images were run on the same gel/immunoblot, with colored lines above corresponding to groups displayed in bar graph. Data represented as mean ± standard error of the mean (SEM). Data normalized to naïve cells without acute insulin stimulation. Data analyzed via two-way Analysis of Variance (ANOVA) with Tukey’s Honest Significant Difference (HSD) post hoc comparison. * = *p* < 0.05. n = 9, N = 2. HI = hyperinsulinemic.

Upon insulin stimulation, nitric oxide (NO) production in endothelial cells is mediated by the phosphatidylinositol-3-kinase/protein kinase B (PI3K/AKT) pathway [30]. We previously showed that this pathway is downregulated in hyperinsulinemic primary mouse brain endothelial cells (MBECs) [29]. Given the importance of NO in insulin-mediated vasoregulation, we first sought to assess NO production capacity in MBECs (n = 9, N = 2). Endothelial nitric oxide synthase (eNOS), the enzyme responsible for NO generation, becomes activated by the insulin/PI3K/AKT axis via phosphorylation of the serine 1177 residue (peNOS^S1177^) [30]. peNOS^S1177^/eNOS levels were significantly affected by experimental condition (naïve v. hyperinsulinemic) (*p* = 0.0236, F (1, 32) = 5.653). Acute insulin stimulation greatly reduced the amount of peNOS^S1177^/eNOS in hyperinsulinemic MBECs as compared to naïve (*p* = 0.0105, 44.50% difference; Figure 1A,B). These data support that hyperinsulinemia impairs NO production capability in MBECs.

### 2.2. Insulin Receptor Internalization into MBECs Is Impaired with Hyperinsulinemia

**Figure 2:**
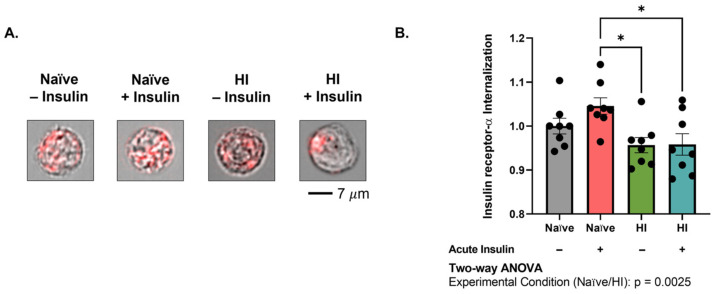
Hyperinsulinemia impairs insulin receptor internalization into brain microvascular endothelial cells. (**A**) Representative images and (**B**) quantification of insulin receptor-α (red) internalization in relation to cell boundary (brightfield). Images are representative of insulin receptor-α internalization in reference to cell boundary, not fluorescence/brightfield intensity. Data represented as mean ± standard error of the mean (SEM). Data normalized to naïve cells without acute insulin stimulation. Data analyzed via two-way Analysis of Variance (ANOVA) with Tukey’s Honest Significant Difference (HSD) post hoc comparison. * = *p* < 0.05. n = 8, N = 2. HI = hyperinsulinemic.

We previously reported that hyperinsulinemia alters insulin receptor presentation and reduces downstream insulin signaling in MBECs. Interestingly, we observed an increase in both total and membrane-bound insulin receptor levels in MBECs prior to acute insulin stimulation [29]. As insulin signaling is tightly linked to internalization, we aimed to directly compare insulin receptor internalization in naïve and hyperinsulinemic MBECs with and without acute insulin stimulation (n = 8, N = 2). A two-way Analysis of Variance (ANOVA) revealed a significant effect of experimental condition on internalization (*p* = 0.0025, F (1, 28) = 10.98). Importantly, Tukey’s Honest Significant Difference (HSD) post hoc comparison identified that acute insulin stimulation significantly reduced internalization in hyperinsulinemic MBECs as compared to naïve cells (*p* = 0.0199, 8.7% difference; Figure 2A,B). These results confirm that acute insulin stimulation impairs insulin receptor internalization following hyperinsulinemic conditioning. 

### 2.3. Clathrin-Mediated Mechanisms Promote Insulin Receptor Internalization into MBECs and Become Impaired with Hyperinsulinemia

**Figure 3:**
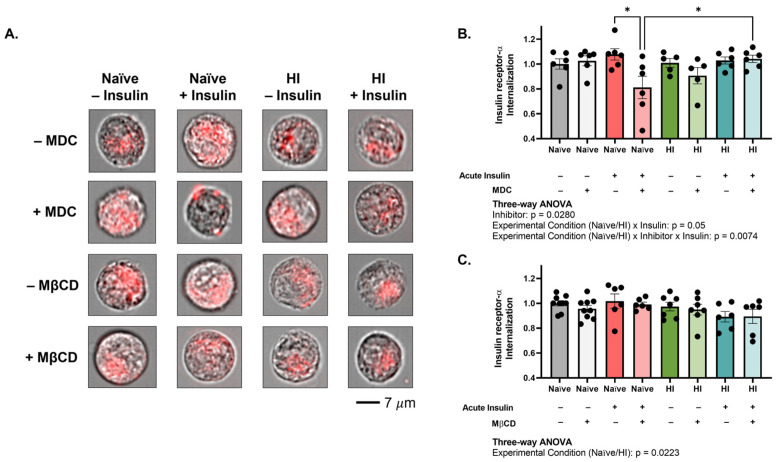
Clathrin-mediated endocytosis is responsible for insulin receptor internalization following acute insulin stimulation only into healthy brain microvascular endothelial cells. (**A**) Representative images and quantification of insulin receptor-α (red) internalization in relation to cell boundary (brightfield) following pharmacologic inhibition of (**B**) clathrin-mediated endocytosis (monodansylcadaverine; MDC) and (**C**) caveolin-mediated endocytosis (methyl-β-cyclodextrin, MβCD). Images are representative of insulin receptor-α internalization in reference to cell boundary, not fluorescence/brightfield intensity. Data represented as mean ± standard error of the mean (SEM). Data normalized to naïve cells without acute insulin stimulation nor inhibitor treatment. Data analyzed via three-way Analysis of Variance (ANOVA) with Tukey’s Honest Significant Difference (HSD) post hoc comparison. * = *p* < 0.05. n = 5–6, N = 2. HI = hyperinsulinemic.

Insulin receptor can be internalized by both clathrin- or caveolin-dependent mechanisms on a tissue-specific basis [24,25,26]. To better contextualize the impact of hyperinsulinemia on internalization, we next sought to determine the mechanism by which the insulin receptor is internalized into MBECs (n = 5–6, N = 2). Blockade of clathrin-mediated endocytosis by monodansylcadaverine (MDC) significantly impaired insulin receptor internalization across experimental conditions and insulin treatments (interaction: *p* = 0.0074, F (1, 38) = 8.023), with the effect specifically observed in naïve MBECs following acute insulin stimulation (*p* = 0.0113, 28.13% difference; Figure 3A,B). Insulin receptor internalization was significantly affected only by experimental condition (*p* = 0.0223, F (1, 48) = 5.577) following inhibition of caveolin-mediated endocytosis by methyl-β-cyclodextrin (MβCD; Figure 3A,C), with no post hoc differences observed. These data suggest that the insulin receptor is internalized into healthy MBECs via a clathrin-mediated mechanism upon acute insulin stimulation. Unlike their naïve counterparts, insulin receptor internalization specifically into hyperinsulinemic MBECs, with and without acute insulin stimulation, was not significantly affected by MDC-mediated clathrin inhibition, nor MβCD-mediated caveolin inhibition (Figure 3A–C). Interestingly, there was a significant increase in internalization in MDC-treated hyperinsulinemic cells as compared to MDC-treated naïve cells following acute insulin stimulation (*p* = 0.0420, 24.86% difference; Figure 3A,B), suggesting that clathrin-mediated endocytosis is not utilized for insulin receptor internalization in conditions of hyperinsulinemia.

**Figure 4:**
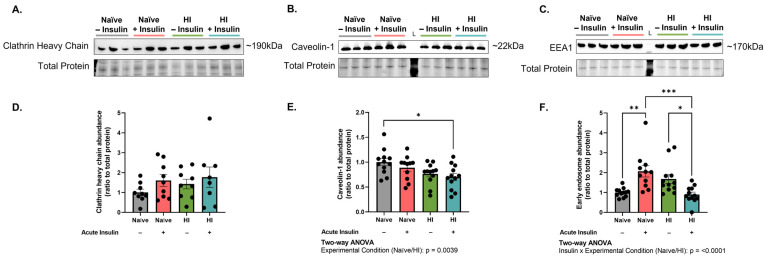
Acute insulin stimulation of hyperinsulinemic brain endothelial cells reduces early endosome availability without promoting significant changes in endocytic protein levels. (**A**–**C**) Western immunoblot images and (**D**–**F**) densitometric quantification of (**A**,**D**) clathrin heavy chain, (**B**,**E**) caveolin-1, and (**C**,**F**) early endosome antigen-1 (EEA1). All chemiluminescent signals were normalized to total protein depicted in the stain-free gel. Groups shown in representative images were run on the same gel/immunoblot, with colored lines above corresponding to groups displayed in bar graph. Data represented as mean ± standard error of the mean (SEM). Data normalized to naïve cells without acute insulin stimulation. Data analyzed via two-way Analysis of Variance (ANOVA) with Tukey’s Honest Significant Difference (HSD) post hoc comparison. * = *p* < 0.05, ** = *p* < 0.01, *** = *p* < 0.001. n = 8–12, N = 2. HI = hyperinsulinemic. L = protein ladder.

To further contextualize these findings, we next assessed the abundance of clathrin heavy chain and caveolin-1 in MBECs (n = 8–12, N = 2). No significant effects were observed in regard to clathrin abundance (Figure 4A,D). An experimental condition effect was observed in regard to caveolin-1 abundance (*p* = 0.0039, F (1, 43) = 9.336), but post hoc testing did not reveal any difference between naïve and hyperinsulinemic cells before or after acute insulin stimulation, respectively (Figure 4B,E). Together, these data suggest that hyperinsulinemia impairs clathrin-mediated insulin receptor endocytosis, an event that is not rescued by caveolin-mediated mechanisms.

### 2.4. Hyperinsulinemia Reduces the Abundance of Downstream Trafficking Centers, Specifically after Insulin Stimulation

The early endosome is a vesicular organelle that serves as the initial sorting hub for newly internalized vesicles [31]. Thus, we next decided to assess early endosome antigen-1 (EEA1) abundance in MBECs (n = 12, N = 2). EEA1 levels were significantly altered by experimental condition and insulin treatment (interaction *p* ≤ 0.0001, F (1, 43) = 23.95). Acute insulin stimulation of naïve MBECs significantly increased EEA1 levels as compared to their basal state (*p* = 0.0016, 69.55% difference) and acutely stimulated, hyperinsulinemic MBECs (*p* = 0.0005, 78.79% difference). Interestingly, acute insulin stimulation of hyperinsulinemic MBECs also reduced EEA1 levels as compared to basal counterparts (*p* = 0.0246, 60.68% difference; Figure 4C,F). As demonstrated, hyperinsulinemia worsens insulin receptor internalization into MBECs. These results further indicate that acute insulin stimulation alters downstream trafficking centers in hyperinsulinemic MBECs. 

### 2.5. Stimulation of Hyperinsulinemic Cells with Insulin Disrupts the Phosphorylation Balance of Insulin Receptor Substrate 1 (IRS_1_) Residues Needed for Insulin Receptor Internalization

**Figure 5:**
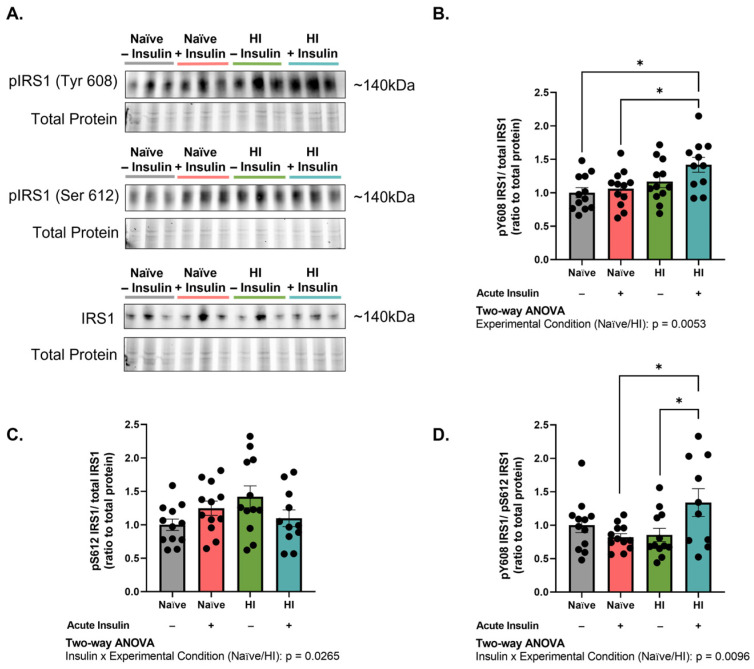
Acute insulin stimulation of hyperinsulinemic brain endothelial cells amplifies tyrosine phosphorylation of insulin receptor substrate 1 (IRS_1_). (**A**) Western immunoblot images and (**B**–**D**) densitometric quantification of (**B**) IRS_1_ phosphorylated at tyrosine 608 (pIRS_1_^Y608^) in relation to total IRS_1_, (**C**) IRS_1_ phosphorylated at serine 612 (pIRS_1_^S612^) in relation to total IRS_1_, and (**D**) pIRS_1_^Y608^/pIRS_1_^S612^ ratio. All chemiluminescent signals were normalized to total protein depicted in the stain-free gel. Groups shown in representative images were run on the same gel/immunoblot, with colored lines above corresponding to groups displayed in bar graph. Data represented as mean ± standard error of the mean (SEM). Data normalized to naïve cells without acute insulin stimulation. Data analyzed via two-way Analysis of Variance (ANOVA) with Tukey’s Honest Significant Difference (HSD) post hoc comparison. * = *p* < 0.05. n = 10–12, N = 2.

Balanced signaling through insulin receptor substrate 1 (IRS_1_) is crucial for clathrin-mediated endocytosis of the insulin receptor. Namely, concomitant dephosphorylation of IRS_1_ tyrosine 608 (pIRS_1_^Y608^; equivalent to human IRS_1_ tyrosine 612) and phosphorylation of IRS_1_ serine 612 (pIRS_1_^S612^; equivalent to human IRS_1_ serine 616) is important for proper recruitment of clathrin to the site [26]. Thus, we assessed the phosphorylation status of pIRS_1_^Y608^ and pIRS_1_^S612^ in MBECs (n = 10–12, N = 2). pIRS_1_^Y608^/IRS_1_ levels were significantly affected by experimental condition (*p* = 0.0053, F (1, 43) = 8.610). Post hoc testing identified that acute insulin stimulation increased pIRS_1_^Y608^/IRS_1_ levels in hyperinsulinemic as compared to naïve MBECs (*p* = 0.0377, 28.70% difference; Figure 5A,B). pIRS_1_^S612^/IRS_1_ levels were affected by experimental condition and insulin treatment (interaction *p* = 0.0265, F (1, 43) = 5.281) (Figure 5A,C). A significant interaction was also observed in the pIRS_1_^Y608^/pIRS_1_^S612^ ratio (*p* = 0.0096, F (1, 42) = 7.371). Acute insulin stimulation significantly increased the pIRS_1_^Y608^/pIRS_1_^S612^ ratio in hyperinsulinemic cells as compared to naïve (*p* = 0.0270, 48.04% difference; Figure 5D). These data support that acute insulin stimulation of hyperinsulinemic MBECs promotes aberrant phosphorylation of IRS_1_ residues involved in clathrin-mediated endocytosis. 

### 2.6. Hyperinsulinemia Reduces Src Homology Phosphatase 2 (SHP2) Activity and Disrupts the SHP2-IRS_1_ Binding Site

**Figure 6:**
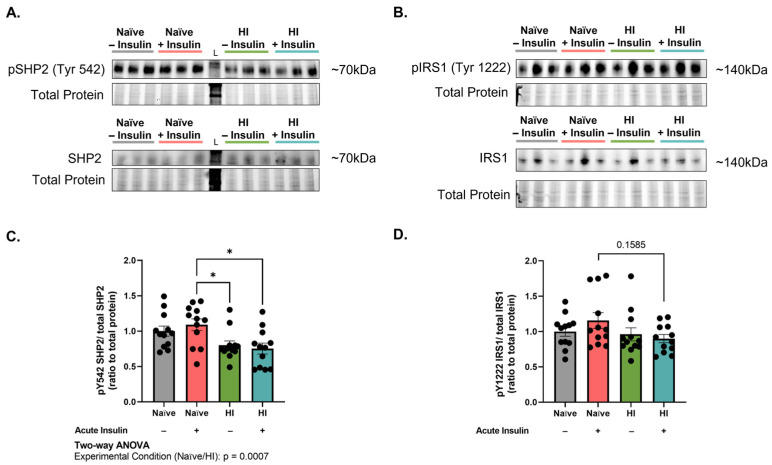
Hyperinsulinemia reduces Src homology phosphatase 2 (SHP2) activity. (**A**,**B**) Western immunoblot images and (**C**,**D**) densitometric quantification of (**A**,**C**) SHP2 phosphorylated at tyrosine 542 (pSHP2^Y542^) in relation to total SHP2 and (**B**,**D**) insulin receptor substrate 1 (IRS_1_) phosphorylated at tyrosine 1222 (pIRS_1_^Y1222^) in relation to total IRS_1_. All chemiluminescent signals were normalized to total protein depicted in the stain-free gel. Groups shown in representative images were run on the same gel/immunoblot, with colored lines above corresponding to groups displayed in bar graph. IRS_1_ representative image the same as in Figure 5. Data represented as mean ± standard error of the mean (SEM). Data normalized to naïve cells without acute insulin stimulation. Data analyzed via two-way Analysis of Variance (ANOVA) with Tukey’s Honest Significant Difference (HSD) post hoc comparison. * = *p* < 0.05. n = 12, N = 2. HI = hyperinsulinemic. L = protein ladder.

Dephosphorylation of pIRS_1_^Y608^ is mediated by the tyrosine phosphatase, Src homology phosphatase 2 (SHP2) [26]. Thus, given the observed increase in IRS_1_^Y608^/IRS_1_ levels in hyperinsulinemic MBECs, we next chose to examine the activity of the IRS_1_^Y608^ phosphatase, SHP2 (n = 12, N = 2). Acute insulin stimulation is known to activate SHP2 via phosphorylation of the tyrosine 542 residue (pSHP2^Y542^) [32]. pSHP2^Y542^/SHP2 levels were significantly affected by experimental condition (*p* = 0.0007, F (1, 44) = 13.33). Notably, acute insulin stimulation reduced pSHP2^Y542^/SHP2 levels in hyperinsulinemic MBECs as compared to naïve (*p* = 0.0116, 36.59% difference; Figure 6A,C). We further observed a trending decline in IRS_1_ tyrosine 1222 (IRS_1_^Y1222^) phosphorylation, one of the docking sites for SHP2, following acute stimulation of hyperinsulinemic cells as compared to naïve MBECs (*p* = 0.1585; Figure 6B,D). Thus, these data suggest that SHP2 action contributes to the aberrant phosphorylation of IRS_1_ residues involved in clathrin-mediated endocytosis in hyperinsulinemic MBECs.

## 3. Discussion

The brain is a highly metabolic organ, requiring roughly 20% of the cardiac output for the body to support its function [33]. As such, appropriate cerebral perfusion is tightly regulated and necessary for optimal brain health [22]. A large body of evidence supports the role of insulin in blood flow regulation, given its direct action on endothelial cells to produce nitric oxide [13]. These molecular observations notably translate to the clinical sphere, as patients with a stronger resistance to insulin display impairments in cerebral perfusion at both the macro- and micro-vascular levels [21]. As such, we first aimed to assess the impact of hyperinsulinemia on brain endothelial nitric oxide production capacity. The activation of endothelial nitric oxide synthase was significantly impaired in hyperinsulinemic cells following acute insulin stimulation (Figure 1), which supports the notion that hyperinsulinemia alters the regulation of molecular targets involved in cerebral blood flow. 

Insulin induced nitric oxide production is a direct result of signaling through the insulin receptor. Proper insulin signaling relies on the internalization of insulin bound to the insulin receptor [23,26,34]. Accelerated internalization can reduce insulin sensitivity due to a lesser number of receptors available for ligand binding. Contrarily, impaired internalization can reduce insulin sensitivity by prolonging the exposure time of receptors to ligand, thereby promoting desensitization [23]. Previous studies investigated the mechanisms of insulin internalization at the level of the blood–brain barrier, with varying results [14,35]. Here, we sought to contextualize the role of hyperinsulinemia in aberrant insulin receptor internalization into brain endothelial cells. Our results indicate that insulin receptor is normally taken up into brain endothelial cells via clathrin-mediated internalization (Figure 2 and Figure 3), a finding consistent with a recent report by Pemberton, et al. implicating clathrin in insulin surface binding and signal regulation in brain microvessels [35]. Gray, et al., however, previously identified lipid raft-mediated endocytosis as the driver of insulin uptake into rat brain microvascular endothelial cells [14]. It is probable that this discrepancy primarily rests in the focus on insulin versus the insulin receptor. Although the carrier involved in insulin blood–brain barrier (BBB) transcytosis remains controversial, insulin receptors constitute a major pathway in the propagation of insulin signaling cascades [36]. Thus, as our analysis focuses specifically on the insulin receptor, we are reducing the possibility of a confounding crosstalk between endothelial transport and signaling pathways. The concentration of insulin employed may further explain this discrepancy. Gray, et al. assessed insulin uptake in endothelial cells following exposure to 200 pM of radioactive insulin in culture, 100-fold less than our acute stimulation parameters (20 nM) [14]. The amount of insulin available may alter the biological activity and processing of the receptor itself, leading to the conflicting results.

Our results demonstrate that inhibition of clathrin-mediated endocytosis did not alter internalization in hyperinsulinemic cells as in naïve cells (Figure 3A,B), suggesting that hyperinsulinemia reduces the role of clathrin in insulin receptor uptake into brain endothelial cells. We acknowledge that naïve cells displayed a reduction (~30%) in internalization following clathrin-inhibition which is consistent with findings in the literature [37]. Although it has been proposed that caveolin-mediated mechanisms may supplement in cases of clathrin impairment in microvessels [35], our inhibitor data suggest that this is not the case in our in vitro model of hyperinsulinemia. Given the observed reduction in insulin receptor internalization in hyperinsulinemic brain endothelial cells, we hypothesize that compensation to an alternative internalization mechanism following acute insulin stimulation is limited. However, further investigation into these compensatory pathways, such as macropinocytosis, may be warranted. 

Given the observed impairment in clathrin-mediated internalization following hyperinsulinemia, we sought to better understand the mechanics underlying this shutdown. In peripheral cells, clathrin-mediated internalization of the insulin receptor is reliant on the balanced phosphorylation of the insulin signaling effector, IRS_1_. Upon insulin binding the insulin receptor, propagation of the insulin signaling cascade results in serine phosphorylation and tyrosine dephosphorylation in the effector binding region of the protein, allowing for the recruitment of clathrin and associated adaptor molecules to the site of internalization. Importantly, dephosphorylation of IRS_1_^Y608^ allows for proper molecular interactions of IRS_1_ and the clathrin adaptor, AP2 [26,34]. Molecular modeling has proposed that phosphorylation at IRS_1_^Y608^ promotes steric hinderance at the site, disrupting the interaction and preventing clathrin-mediated endocytosis [26]. Our data indicate that acute insulin stimulation following hyperinsulinemia significantly increases the phosphorylation of IRS_1_^Y608^ (Figure 5), suggesting that clathrin-mediated mechanisms are prevented due to a lack of molecular engagement with respective players at the internalization site. Phosphorylation and conformational opening of AP2 additionally influence its clathrin-binding capabilities [38]. Given the known signaling dysfunction imparted by hyperinsulinemia [29], additional investigation into AP2 binding kinetics and phosphorylation status should be addressed in future studies to fully characterize its role in our cell model. 

We demonstrated that the increased phosphorylation in IRS_1_^Y608^ following hyperinsulinemia and acute insulin stimulation occurs in the setting of reduced activity of its tyrosine phosphatase, SHP2 (Figure 6). Pharmacologic SHP2 inhibition has been previously reported to impair insulin receptor internalization both in vivo and in vitro, supporting our findings [39]. It is unclear, however, why IRS_1_^Y608^ phosphorylation occurs in the first place. Following insulin binding and autophosphorylation, the insulin receptor is known to phosphorylate IRS_1_^Y608^ [26]. Our previous work indicates impaired insulin receptor activity in hyperinsulinemic MBECs following acute insulin stimulation [29]. We postulate that two scenarios may account for this discrepancy. First, IRS_1_^Y608^ phosphorylation may not be solely reliant on insulin receptor activity. Insulin can bind to insulin-like growth factor receptors (IGF-R), albeit at a lower affinity. Insulin signaling through insulin receptors and IGF-R follow similar paths, including the phosphorylation of IRS_1_ [40]. Thus, it is possible that IGF-R activity may contribute to this observation. Second, acute insulin stimulation following hyperinsulinemia reduces, but does not eliminate, insulin receptor activity [29]. Therefore, although IRS_1_ phosphorylation may occur at a lesser rate, the remnant signal increase in tandem with reduced SHP2 activation and docking capabilities may result in this observation. To our knowledge, there are currently no other kinases known to phosphorylate IRS_1_ at this given residue. Thus, future studies into the regulation of IRS_1_^Y608^ are warranted.

We observed an interesting relationship between SHP2 activity and IRS_1_^Y608^ in hyperinsulinemic cells with and without acute insulin stimulation. In hyperinsulinemic cells following acute insulin stimulation, IRS_1_^Y608^ phosphorylation expectedly increased in the setting of reduced SHP2 activation. However, in hyperinsulinemic cells without acute insulin stimulation, phosphorylation of both IRS_1_^Y608^ and SHP2^Y542^ were near or below baseline. We suspect that this observation is due to desensitization of the cells following 12 h in culture. With no acute stimulation to re-invigorate the insulin signaling cascade, basal signaling through the insulin receptor and, consequently, IRS_1_^Y608^ phosphorylation would likely be low. The basal reduction in SHP2^Y542^ phosphorylation as compared to naïve cells in their basal state is intriguing and would require additional investigation. 

A study by Talbot, et al. that investigated insulin resistance in brain slices of dementia patients provides a clinical perspective on our results. The authors discovered that basal levels of IRS_1_^Y612^ (human correlate to the mouse IRS_1_^Y608^) and IRS_1_^S616^ (human correlate to the mouse IRS_1_^S612^) were elevated in the hippocampus of dementia brains as compared to healthy controls [41]. We observed a similar trending increase in the IRS_1_^S612^ residue in unstimulated, hyperinsulinemic cells compared to naïve (Figure 5C). Acute stimulation, with 1 nM of insulin, dephosphorylated both residues, contrary to our results depicting increased IRS_1_^Y608^ with insulin stimulation. This discrepancy likely rests on study participants having no history of metabolic disease and the focus on neurons as opposed to the vascular endothelium [41]. Notwithstanding, this study and ours synergistically support the notion that aberrant IRS_1_ phosphorylation is intimately linked to cognitive performance and disease. 

In conclusion, we present support for a mechanism by which hyperinsulinemia impairs insulin receptor internalization into brain endothelial cells, thereby altering the regulation of molecular targets involved in cerebral blood flow. Use of a monolayer in vitro culture system has allowed our group to identify the isolated effects of hyperinsulinemia on insulin homeostasis within brain endothelial cells. Given the contribution of various cell types to the overall functioning of the BBB [42], a notable limitation to our study is the use of a single cell-type culture. Thus, it is necessary for future studies to employ more robust models. For example, insulin receptor expression in astrocytes has been shown to influence insulin transport [43]. Thus, an in vitro co-culture system that consists of endothelial cells and astrocytes would better recapitulate the complexity of the BBB and allow for a more robust investigation into the nuances of insulin receptor dynamics. An in vivo model of diet-induced obesity could also be employed to better characterize insulin receptor internalization within the brain microvasculature and cerebral blood flow. These pre-clinical results would provide crucial insight within a complete BBB system, paving the way for potential clinical translation. 

Although controversial as to whether the insulin receptor transports insulin across the BBB [17,18], our results provide context to the mechanisms underlying insulin receptor internalization. Should additional studies validate its role in the transport of insulin into the brain, our findings can be applied in the study of this biological process. The results of our work are significant in that we have identified pathophysiologic disturbances in molecular targets associated with the regulation of insulin signal propagation and cerebral blood flow, the likes of which could possibly be used in the repurposing or development of therapeutics to help combat VCID. 

## 4. Materials and Methods

### 4.1. Microvascular Endothelial Cell Culture 

**Figure 7:**
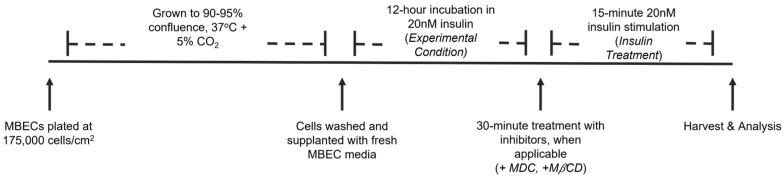
Outline of culturing and stimulation parameters.

Primary C57BL/6 mouse brain microvascular endothelial cells (MBECs; C57-6023, Cell Biologics, Chicago, IL, USA) were cultured as per our previously established protocol [29]. In brief, MBECs were seeded at a density of 175,000 cells/cm^2^ into 12 well plates coated with gelatin-based coating solution (Cell Biologics). Cells were grown in complete mouse endothelial cell medium with supplement (Cell Biologics) to ~90–95% confluence at 37 °C and 5% CO_2_. Cells were incubated for 12 h overnight in 20 nM human recombinant insulin (Sigma, St. Louis, MO, USA) to induce basal hyperinsulinemia. Selection of incubation time and insulin concentration for hyperinsulinemic conditioning and acute insulin stimulation was dictated by pilot studies as previously described [29]. Acute insulin stimulation was subsequently performed, with protocol-specific indications described in the following sections. Figure 7 outlines culturing parameters and relevant terminology. All experiments were completed on passage 5. Human recombinant insulin was diluted directly into culture media. 

### 4.2. Flow Cytometry

Cells were incubated for 11.5 h overnight in 20 nM human recombinant insulin to promote hyperinsulinemia. Naïve and hyperinsulinemic overnight media was collected into centrifuge tubes and kept warm at 37 °C. Cells were washed 3× in 1× phosphate buffered saline (PBS) (Corning™ Mediatech™, Tewksbury, MA, USA) and detached following a 2 min incubation with warmed 0.05% trypsin-EDTA (Gibco, Billings, MT, USA). Fresh media was added to neutralize the trypsin and cells were spun at 2000 rpm for 5 min. Pelleted cells were resuspended in naïve or hyperinsulinemic media, corresponding to the treatment received, and placed at 37 °C for 30 min to recover from trypsinization (equating to 12 h total of hyperinsulinemic treatment prior to processing). Remaining naïve and hyperinsulinemic media was chilled during this time. Cells were washed 1× in ice-cold fluorescence-activated cell sorting (FACS) buffer (1× PBS with 2% fetal bovine serum, FBS) and resuspended in the now chilled corresponding media containing a polyclonal antibody against insulin receptor-α at a 1:20 dilution (Alexa Fluor 647 conjugated; Bioss Antibodies, Woburn, MA, USA). Cells were incubated on ice in the dark for 40 min and subsequently washed 2× in ice-cold FACS buffer. Cells were stimulated with 20 nM human recombinant insulin or vehicle control for 15 min at 37 °C. Immediately following insulin/vehicle stimulation, cells were fixed in 4% paraformaldehyde at 4 °C for 10 min. Cells were then washed 3× in ice-cold FACS buffer, filtered through a 40 μM mesh, and immediately run on the Amnis ImageStream^X^ Mk II platform (Luminex Corporation, Austin, TX, USA). One-thousand events were collected for each sample run. All data were processed on the IDEAS version 6.2 platform (Luminex Corporation) using the Internalization Wizard. Insulin receptor-α internalization was assessed in relation to the brightfield cell boundary [44]. Values greater than 0 indicate enhanced internalization, while values less than 0 indicate enhanced membrane presence. 

### 4.3. Endocytosis Inhibitor Treatment

Immediately preceding insulin stimulation in flow cytometry preparations, cells were pre-treated with monodansylcadaverine (MDC) (20 μM, DMSO, Sigma), methyl-β-cyclodextrin (MβCD) (10 mM, culture media, Sigma), or relevant vehicle controls for 30 min at 37 °C [45,46,47]. MDC inhibits clathrin-mediated endocytosis by stabilizing clathrin-coated vesicles. MβCD disrupts caveolae by stripping cholesterol from the membrane and disrupting lipid rafts [48]. DMSO concentration was maintained at <0.5% in both experimental and vehicle treated samples.

### 4.4. Western Immunoblotting

Naive and hyperinsulinemic cells were processed for Western immunoblotting as previously described [29]. Briefly, cells were washed in warm 1× PBS and supplanted with fresh media. Cells were stimulated with 20 nM human recombinant insulin for 15 min at 37 °C. Following insulin stimulation, media was removed, and cells were quickly washed in ice-cold 1× PBS. Cells were then scraped into tissue-protein extraction reagent (T-PER; Thermo Scientific™, Waltham, MA, USA) with added protease (Roche Diagnostics, Indianapolis, IN, USA) and phosphatase (Pierce™, Waltham, MA, USA) inhibitors and stored at −80 °C until use. At the time of analysis, cell homogenates were heated with sodium dodecyl sulfate (SDS) to create lysates that were subsequently run on SDS-PAGE. Separated samples were transferred to a nitrocellulose membrane and blocked for 1 h at room temperature in 5% milk-1× tris-buffered saline (TBS) + Tween-20. Antibodies were diluted into 5% milk- or 5% bovine serum albumin (BSA)-1× TBS + Tween-20 as per the manufacturer’s instructions. Antibodies against clathrin heavy chain, caveolin-1, early endosome antigen-1 (EEA1), Src homology phosphatase 2 (SHP2), SHP2 phosphorylated at tyrosine residue 542 (pSHP2^Y542^), endothelial nitric oxide synthase (eNOS), and eNOS phosphorylated at serine residue 1177 (peNOS^S1177^) were used at a 1:1000 dilution. Insulin receptor substrate (IRS_1_), IRS_1_ phosphorylated at tyrosine residue 608 (pIRS_1_^Y608^, Invitrogen, Waltham, MA), IRS_1_ phosphorylated at serine residue 612 (pIRS_1_^S612^), and IRS_1_ phosphorylated at tyrosine residue 1222 (pIRS_1_^Y1222^) were used at a 1:750 dilution (predicted ~130 kDa, ~apparent molecular weight 160 kDa–180 kDa, observed ~140 kDa). Relevant secondary antibodies (Abcam, Cambridge, United Kingdom) conjugated to horseradish peroxidase were used at a 1:2000 dilution. All antibodies were from Cell Signaling Technology (Danvers, MA, USA) unless otherwise indicated. Wash steps were performed in 1× TBS + Tween-20. Protein signals were visualized with Clarity Western enhanced chemiluminescence (ECL) Substrate (Bio-Rad Laboratories, Hercules, CA, USA). Both stain-free gel and chemiluminescent signals were imaged on the ChemiDoc^TM^ imaging platform (Bio-Rad Laboratories). All data were processed in the ImageLab software, version 6.1.0 (Bio-Rad Laboratories), and chemiluminescent signals were normalized to total protein concentrations per lane according to the manufacturer’s instructions.

### 4.5. Statistical Analysis

Statistical analyses were performed using Prism, version 9.3.1 (GraphPad Software, Inc., San Diego, CA, USA). Data from each individual experiment were normalized to a basal naïve internal control (baseline). All data were analyzed via a two-way Analysis of Variance (ANOVA), except for data represented in Figure 3 (three-way ANOVA). Tukey’s Honest Significant Difference (HSD) was employed to assess multiple comparisons between groups. All data are represented as mean ± standard error of the mean (SEM). Significance was established at an α level of 0.05. Experiments were run with biological (n) and technical replicates (N) as indicated in figure legends. 

## Data Availability

The data that support the findings of this study are available in https://doi.org/10.6084/m9.figshare.23822538 (accessed on 15 September 2023).
